# p53 sensitizes chemoresistant non-small cell lung cancer via elevation of reactive oxygen species and suppression of EGFR/PI3K/AKT signaling

**DOI:** 10.1186/s12935-019-0910-2

**Published:** 2019-07-19

**Authors:** Yize Zhang, Chae Young Han, Fu Gang Duan, Xing-Xing Fan, Xiao-Jun Yao, Robin J. Parks, Yi-Jun Tang, Mei-Fang Wang, Liang Liu, Benjamin K. Tsang, Elaine Lai-Han Leung

**Affiliations:** 1 State Key Laboratory of Quality Research in Chinese Medicine, Macau Institute for Applied Research in Medicine and Health, Macau University of Science and Technology, Avenida Wai Long, Taipa, Macao, China; 2 Departments of Obstetrics & Gynecology and Cellular & Molecular Medicine, Ottawa Hospital Research Institute, University of Ottawa and Chronic Disease Program, Ottawa, ON K1H 8L6 Canada; 3 Department of Medicine and Biochemistry, Microbiology & Immunology, Ottawa Hospital Research Institute, University of Ottawa and Regenerative Medicine Program, Ottawa, ON K1H 8L6 Canada; 4 Department of Respiratory and Critical Care, Taihe Hospital, Hubei University of Medicine, Shiyan, Hubei China

**Keywords:** Non-small cell lung cancer (NSCLC), Cis-diaminedichloroplatinum (Cisplatin) (CDDP), Chemoresistance, Epidermal growth factor (EGFR), p53 reactive oxygen species (ROS)

## Abstract

**Background:**

Non-small cell lung cancer (NSCLC) is the leading cause of cancer deaths primarily due to chemoresistance. Somatic mutation of *TP5*3 (36%) and epidermal growth factor receptor (*EGFR*; > 30%) are major contributors to cisplatin (CDDP) resistance. Substantial evidence suggests the elevated levels of reactive oxygen species (ROS) is a key determinant in cancer. The elevated ROS can affect the cellular responses to chemotherapeutic treatments. Although the role of EGFR in PI3K/Akt signaling cascade in NSCLC is extensively studied, the molecular link between EGFR and p53 and the role of ROS in pathogenesis of NSCLC are limitedly addressed. In this study, we investigated the role of p53 in regulation of ROS production and EGFR signaling, and the chemosensitivity of NSCLC.

**Methods:**

In multiple NSCLC cell lines with varied p53 and EGFR status, we compared and examined the protein contents involved in EGFR-Akt-P53 signaling loop (EGFR, P-EGFR, Akt, P-Akt, p53, P-p53) by Western blot. Apoptosis was determined based on nuclear morphological assessment using Hoechst 33258 staining. Cellular ROS levels were measured by dichlorofluorescin diacetate (DCFDA) staining followed by flow cytometry analysis.

**Results:**

We have demonstrated for the first time that activation of p53 sensitizes chemoresistant NSCLC cells to CDDP by down-regulating EGFR signaling pathway and promoting intracellular ROS production. Likewise, blocking EGFR/PI3K/AKT signaling with PI3K inhibitor elicited a similar response. Our findings suggest that CDDP-induced apoptosis in chemosensitive NSCLC cells involves p53 activation, leading to suppressed EGFR signaling and ROS production. In contrast, in chemoresistant NSCLC, activated Akt promotes EGFR signaling by the positive feedback loop and suppresses CDDP-induced ROS production and apoptosis.

**Conclusion:**

Collectively, our study reveals that the interaction of the p53 and Akt feedback loops determine the fate of NSCLC cells and their CDDP sensitivity.

**Electronic supplementary material:**

The online version of this article (10.1186/s12935-019-0910-2) contains supplementary material, which is available to authorized users.

## Background

Lung cancer is the leading cause of cancer death [1]. Non-small lung cancer (NSCLC) is most common histological subtypes, accounting for 85% of all lung cancer [2]. In early stage NSCLC, surgical debulking followed by chemotherapy is the most common and effective treatment [3]. In initial chemotherapy of NSCLC, combinations of tyrosine kinase inhibitor (TKI) and Platinum-containing Cis-diammine dichloroplatinum (II) (cisplatin or CDDP) and its analogs (e.g. Carboplatin) are the standard therapeutic regimen [4, 5]. However, many patients develop chemoresistance within 6 months with unknown etiology [6], leading to poor 5-year survival rate (15%). Chemoresistance is a multifactorial phenomenon and caused by dysregulated cellular drug uptake and drug detoxification, accelerated drug metabolism, and high DNA repair. Dysregulation of pro-apoptotic and anti-apoptotic pathways also play a critical role [7]. Since the chemoresistance limits successful treatment outcome, understanding the mechanism of chemoresistance and identification of effective treatment are urgently needed.

Epidermal growth factor receptor (EGFR) gene is highly expressed and frequently mutated in NSCLC. EGFR mutations are predominantly frame shift mutation or point mutation (e.g. L858R in exon 21). EGFR, a transmembrane protein, transfers extracellular signal into cells by activating tyrosine kinase (TK) [8]. TKI, such as gefitinib, targets EGFR through suppression of TK signaling cascade [9–11]. Despite substantial studies, it remains unknown if EGFR mutation contributes to platinum resistance [12]. It is possible that hyper-activation of EGFR and PI3K/Akt signaling impairs the function of the cell cycle factors p21^Cip1^ or Bax, leading to enhanced cell survival and chemoresistance [13, 14].

Mutation of the tumor suppressor gene *TP53* occurs in 34% in NSCLC patients. Frequent smoking is associated with *TP53* mutation and the development of lung cancer [15]. The degradation of p53 in normal cells is regulated through ubiquitination by the E3 ubiquitin ligase mouse double minute 2 (Mdm2) [16, 17]. Cellular stress, such as CDDP treatment, activates and stabilizes p53 via phosphorylation at the sites of Ser 15 and/or Ser 20, subsequently blocking of p53-Mdm2 interaction and suppressing p53 degradation [17, 18].

Recent evidence indicates that p53 regulates cell fate via modulating cellular reactive oxygen species (ROS) [19]. In cancer cells, ROS is activated by multiple factors, including oncogenes, mitochondrial mutations, hypoxia and loss of tumor suppressors [20]. ROS plays an essential role in regulating various signaling pathways [21]. Elevated ROS levels cause genomic instability, activation of oncogenes [e.g. PI3K, mitogen activated protein kinases (MAPK), and hypoxia-inducible factors (HIFs)] and loss of tumor suppressors (e.g. p53) [22, 23]. ROS activates the PI3K/Akt pathway, which is involved in cell survival and proliferation [24]. Liu et al. [25] reported that PI3K/Akt pathway inhibits ROS production by regulating the expression of transcription factor Forkhead Box Protein O1 (Foxo1) and Caspase-3 involved in intrinsic apoptosis.

On the other hand, while ROS-induced DNA damage commonly results in p53 mutations and impaired p53 function, p53 can serve as antioxidants and regulates a range of antioxidant genes [26, 27]. ROS levels are inherently higher in cancer cells than normal cells, suggesting that cancer cells require smaller increment of cellular ROS for the induction of cell death. Therefore, stimulation of ROS in cancer cell has been considered a new strategy for anti-cancer therapy [28, 29]. We have previously demonstrated that ROS is an upstream factor regulating the phosphorylation of EGFR and in turn induces apoptosis in NSCLC [11], although the mechanism(s) involved is not clear. In addition, the level of ROS is higher in NSCLC cell line with a double mutation at the EGFR, offering potential selectivity in therapeutic strategy for lung cancer [28, 30].

To date, the relationship between ROS and EGFR in NSCLC and how EGFR is regulated in chemoresistance remain unknown. In the present study, we have demonstrated for the first time that (a) CDDP down-regulates EGFR, activates p53 and increases ROS production in chemosensitive but not in chemoresistant NSCLC; (b) Akt confers resistance in NSCLC in part by activating EGFR and down-regulating p53 and ROS production, and (c) p53 activation inhibits EGFR signaling and increases ROS production. Our findings support the hypothesis that p53 plays an important role in the feedback regulation of chemosensitivity in NSCLC, and that p53 mutation attenuates CDDP-induced ROS elevation, suppression of EGFR/PI3K/AKT signaling and apoptosis in chemoresistant NSCLC cells. Our studies further suggest that interaction between p53 and Akt feedback loops determine the fate of NSCLC cells and their CDDP sensitivity.

## Methods

### Reagents

RPMI 1640 medium, fetal bovine serum (FBS, Origin: Canada), penicillin, streptomycin were purchased from Thermo scientific (San Diego, CA, USA). Phosphate Buffered Saline (PBS). Cis-diaminedichloroplatinum (CDDP), Hoechst 33258 were purchased from Sigma-Aldrich (St. Louis, MO, USA) and complete mini EDTA-free tablet was purchased from Roche (Laval, QC, Canada). DC protein assay kit was from Bio-Rad (Hercules, CA, USA). Nitrocellulose (NC) membrane was obtained from GE Healthcare (Waukesha, WI, USA). Detailed information of antibodies used in present studies is described in Additional file 1: Table S1. Dichlorofluorescin diacetate (DCFDA) cellular ROS detection assay kit was purchased from Abcam (Cambridge, MA, USA). DN-Akt (triple-A mutated K179A, T308A, and S473A, kinase-dead dominant negative Akt1) and myristoylated Akt (AAkt) were synthesized at the University of Ottawa Adenoviral core facility. p53 and GFP adenoviral constructs were purchased from Applied Biological Materials (Richmond, BC, Canada). All adenoviruses were amplified and purified in the lab of Dr. Robin Parks (Regenerative Medicine Program, Ottawa Hospital Research Institute, Ottawa, Canada).

### Cell culture and drug treatment

Human NSCLC cell lines (A549, H1299, H1650, and H1975) were purchased from ATCC (Manassas, VA, USA) and its detailed information of NSCLC cell lines are stated in Additional file 1: Table S2. A549, H1650, and H1975 cells were originated from lung adenocarcinoma, harboring wild type p53 gene, while H1299 cells were derived from lung adenocarcinoma with p53 gene mutation (p53-Null). All cell lines were cultured in RPMI 1640 medium contained FBS (10%), penicillin (50 µg/ml) and streptomycin (50 µg/ml). Cells were maintained at 37 °C in a humidified atmosphere with 5% CO_2_.

### Protein extraction and Western blot analysis

Protein extraction and Western blot (WB) analysis were carried as previously reported [31–33]. Cells were lysed with RIPA lysis buffer containing a cocktail of proteinase and phosphatase inhibitors. Samples were incubated (0 °C, 20 min) and centrifuged (12,000*g*, 10 min, 4 °C). Protein concentration was measured by DC protein assay kit and the protein lysate was boiled (5 min, 4× loading buffer). Equivalent amounts of total protein were loaded onto 8–10% acrylamide gels, resolved by electrophoresis (108 V, 2 h) and subsequently electro-transferred on nitrocellulose membranes (300 mA, 2 h). Each membrane was then blocked (1 h; room temperature, RT) with 5% skim milk in TBST and then was immunoblotted (overnight; 4 °C) with specific primary antibodies at indicated dilutions. After washing (5 times; 1× TBST), appropriate secondary antibodies were added. Signal intensity was quantified using a LI-COR Odyssey scanner (Belfast, ME, USA) or enhanced chemiluminescence kit (ThermoFisher Scientific).

### Assessment of apoptosis

Apoptosis was determined based on nuclear morphology, using Hoechst 33258 staining. At the end of the culture period, attached and floating cells were combined and collected by centrifugation (1000*g*, 5 min). The cell pellets were washed with PBS (10%, 3 times) and incubated in 10% formalin PBS solution containing Hoechst 33258 (12 ng/ml) dye overnight in dark. Apoptosis (nuclear condensation and fragmentation) was assessed by fluorescence microscopy, with all samples blindly labeled and at least 400 cells counted. Results are presented as a percentage of apoptotic cells over a total number of cells.

### Adenovirus infection

After culture and plating, cells were infected with an adenoviral construct (Adv) p53, dominant-negative Akt (DN-Akt), activated Akt (AAkt), and GFP control at various multiplicities of infection (MOI) in serum-free medium (37 °C, 5 h) followed by CDDP treatment. Adv-GFP was used to unify the total concentration of virus in each treatment group. The adenovirus infection efficiency was > 90% and its expression was confirmed by WB.

### Assessment of ROS level

Cellular ROS levels were measured by flow cytometry, using 2′,7′ dichlorofluorescein diacetate (DCFDA) [19]. Cells were plated in 6-well plates, and then subjected to the indicated treatments. Negative and positive controls include pretreatment of cells with *N*-acetyl-l-cysteine (NAC; 1 h) and Tert-Butyl Hydrogen Peroxide (TBHP; 4 h), respectively. Cells were harvested by centrifugation (1000*g*, 5 min). The pellets were washed twice with PBS, and each sample was incubated with 20 μM DCFDA working solution (30 min, 37 °C in the dark). ROS was detected using FC 500 Series flow cytometer (Beckman Coulter, CA, USA) with excitation and emission spectra of 488 nm and 525 nm, respectively, and analyzed with FlowJo Version X software (Tree Star, San Carlos, CA).

### Statistical analysis

All experiments were performed at least three times. All data are presented as mean ± SEM and analyzed using one- or two-way ANOVA, and the Bonferroni post hoc test (Graph Pad, Prism Version 7.0 software; San Diego, CA, USA). Statistical probability (*P*) for significance was expressed as *P < 0.05, **P < 0.01, ***P < 0.001, and *P < 0.05.

## Results

### NSCLC cell lines with different EGFR and p53 mutational status show distinct CDDP sensitivity

**Fig. 1:**
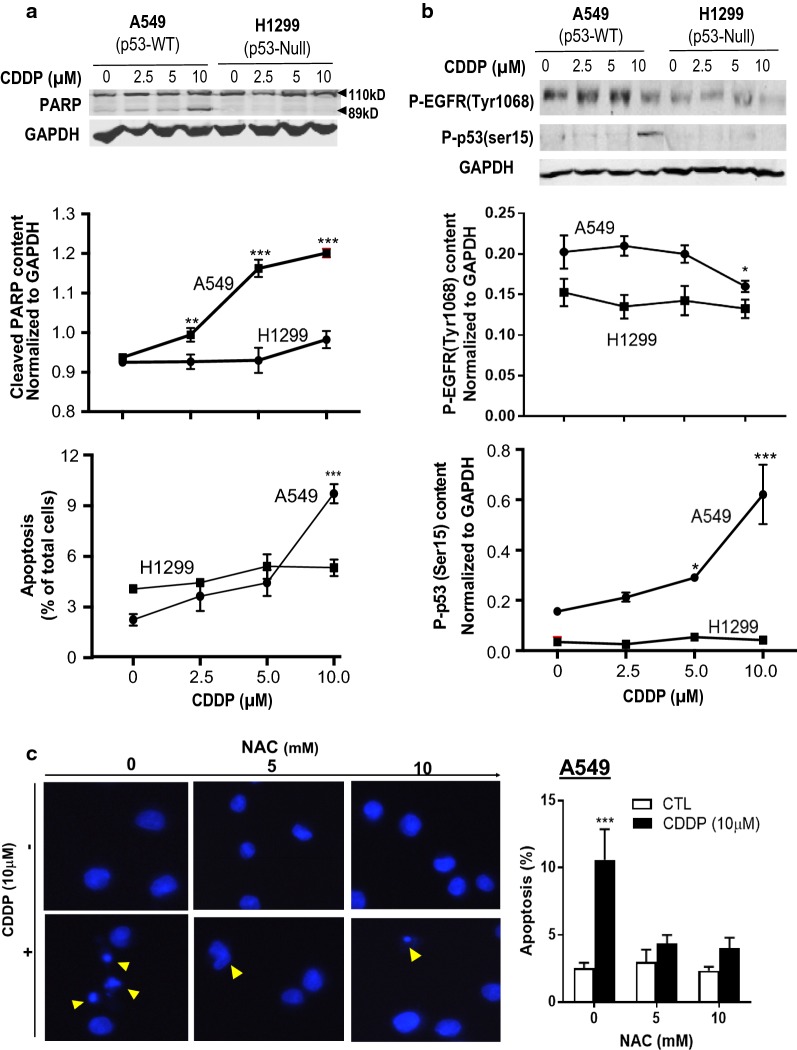
CDDP alters p-EGFR, P-p53 and cleaved-PARP levels and induces ROS-mediated apoptosis in chemosensitive A549 cells but not in chemoresistant H1299 cells. A549 and H1299 cells were cultured with different concentrations of CDDP (0, 2.5, 5 and 10 µM; DMSO as vehicle control) for 24 h. **a** Protein levels of full length PARP (110 kD), cleaved PARP (89 kD) and GAPDH (loading control) were detected by Western blot. Apoptosis was assessed by Hoechst staining. **b** Protein contents of P-EGFR (Try 1068), P-p53 (Ser 15), and GAPDH (loading control) were measured by Western blot. **c** A549 cells were pre-treated with 0, 5 and 10 mM of the reactive oxygen species (ROS) inhibitor *N*-acetyl-l-cysteine (NAC) for 24 h and treated with CDDP (10 µM, 24 h) followed by Hoechst staining. Quantification and statistical analysis of P-EGFR, P-53 (Ser 15) and, cleaved PARP were conducted. All data were presented as mean ± SEM (n = 4). Data were analyzed by two-way ANOVA followed by Bonferroni post hoc test (*P < 0.05, ***P < 0.001; CTL vs. CDDP)

To examine the function of p53 on EGFR activation and CDDP-induced apoptosis, A549 (p53-wt) and H1299 (p53-null) cells, which express wild type EGFR were treated with various concentrations of CDDP (0–10 μM; 24 h) and activated PARP content (shown as PARP cleavage, indicative of caspase 3 activation, 89 kD) and apoptosis were examined. As shown in Fig. 1a, CDDP significantly increased the protein content of cleaved PARP and induced apoptosis in A549 cells, but not in H1299 cells, suggesting that A549 is CDDP-sensitive whereas the opposite is true with H1299 and that a functional p53 is required for CDDP action. We and others have found that CDDP increased phosphorylation of p53 at site of Ser 15 and Ser 20 and stabilizes p53 protein in various cancers, including ovarian cancer, testicular cancer, and sarcoma [34]. Consistent with this finding, CDDP markedly increased P-p53 (Ser 15) protein content and attenuated P-EGFR (Try 1068) activation in A549 cells but not in H1299 cells (Fig. 1b).

To determine if NSCLC cells with different EGFR and p53 mutational status exhibit different CDDP sensitivity, we cultured multiple NSCLC cells (A549, H1299, H1650 and H1975) with CDDP (10 μM; 24 h) and assessed the protein level of cleaved PARP and the apoptosis rate (%) by Western blot (WB) and Hoechst staining, respectively. The mutational status of *EGFR* and *TP53* genes in these cell lines are shown in Additional file 1: Table S2 [35, 36]. As shown in Additional file 1: Fig. S1, CDDP significantly increased cleaved PARP content and apoptosis in A549 and H1650 cells, but not in H1299 cells, suggesting that A549 and H1650 are CDDP sensitive and could undergo apoptosis in response to CDDP while H1299 showed no PARP cleavage and CDDP chemoresistance. Although H1975 showed significant basal level of PARP cleavage in both treated and non-treated groups, no increase was observed with CDDP treatment (Additional file 1: Fig. S1a). Also, these findings are consistent with the apoptotic response to CDDP as determined by nuclear morphology (DNA fragmentation; Additional file 1: Fig. S1b).

To examine whether CDDP-induced apoptosis in chemosensitive NSCLC cell is partly due to ROS, we pre-treated chemosensitive A549 cells with the pharmacological ROS inhibitor, *N*-acetyl-l-cysteine (0, 5, 10 mM, 24 h) and then treated with CDDP (10 µM, 24 h). CDDP-induced apoptosis was significantly decreased in NAC pre-treated cells (Fig. 1c) compared with cells only treated with CDDP, suggesting that CDDP likely caused ROS-induced apoptosis in chemosensitive NSCLC cells.

### p53 sensitizes chemoresistant NSCLC cells to CDDP by suppressing EGFR signaling

**Fig. 2:**
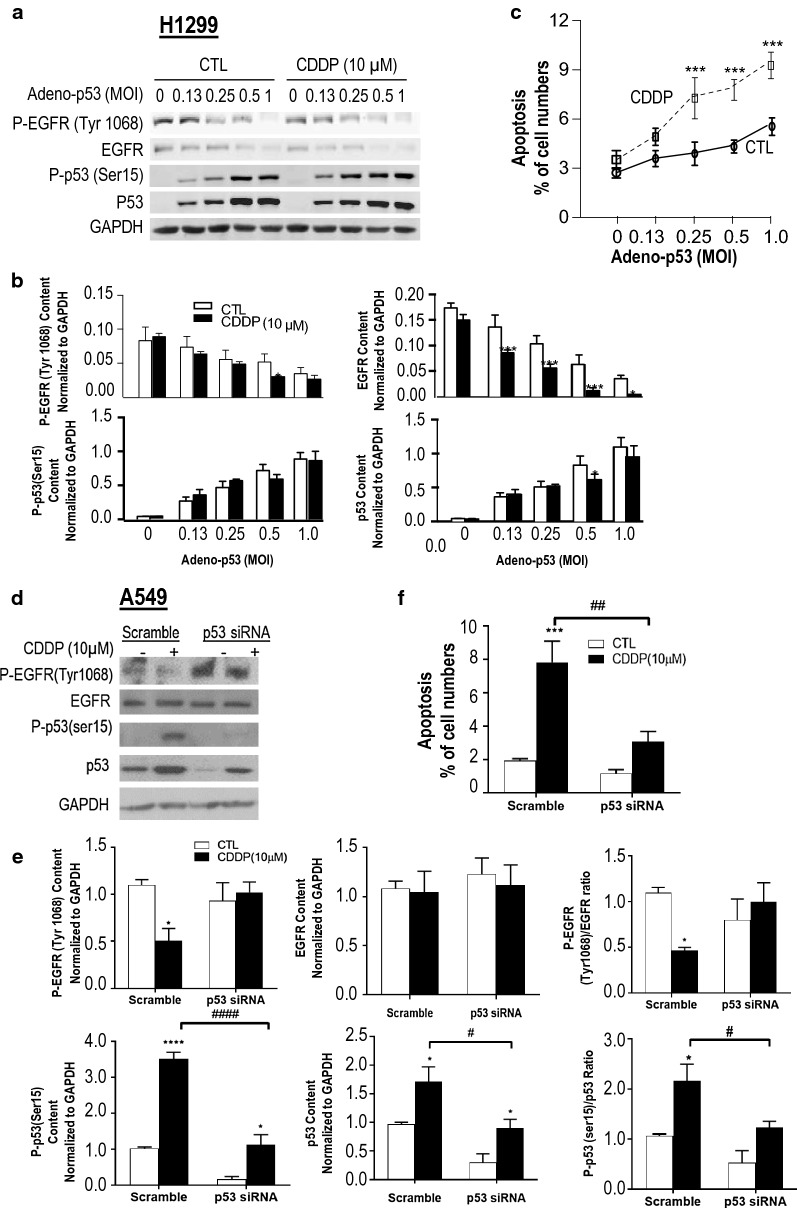
p53 is required for suppression of EGFR activity and CDDP-induced apoptosis in NSCLC cells. Chemoresistant H1299 cells were infected with adeno virus p53 (Adv-p53) (Multiplicity of Infection, MOI = 0, 0.25, 0.5 and 1; 5 h) followed by CDDP treatment (10 μM, 24 h). **a** Protein contents of P-EGFR (Try 1068), EGFR, P-p53 (Ser 15), p53 and GAPDH (loading control) were detected by Western blot. **b** Statistical analysis and quantification of indicated proteins [P-EGFR (Try 1068), EGFR, P-p53 (Ser 15) and p53] were performed. **c** Apoptosis was assessed by Hoechst staining. **d** A549 cells were transfected with either p53 siRNA or its scramble control (100 nM, 16 h) and treated with CDDP (10 µM, 24 h). Protein levels of p-EGFR, EGFR, P-p53, p53, GAPDH (loading control) were detected by Western blot. **e** Quantification and statistical analysis of indicated proteins were conducted. **f** Apoptosis before and after p53 knockdown (siRNA) and CDDP treatment were determined. All data were presented as mean ± SEM (n = 3). Data were analyzed by two-way ANOVA and Bonferroni post hoc test (*P < 0.05, **P < 0.01, ***P < 0.001; CTL vs. CDDP; ^#^*P *< 0.05, ^#^*P *< 0.01, ^####^*P* < 0.0001, Scramble vs. p53 siRNA)

To investigate whether p53 is involved in the regulation of EGFR in NSCLC cells, chemoresistant H1299 (p53 null) cells were infected with adenoviral construct (Adv)-wild-type p53 (Adv-p53) or Adv-GFP (as control) at different multiplicity of infection (MOI: 0–1.0; 5 h), and then treated with CDDP (10 μM, 24 h). As shown in Fig. 2a and b, wild-type p53 reconstitution in chemoresistant p53-null H1299 cells (confirmed by WB) decreased p-EGFR protein content irrespective of the presence of CDDP and facilitated CDDP-induced EGFR down-regulation, suggesting that CDDP significantly down-regulated EGFR in a p53-dependent manner. To determine if CDDP-induced apoptosis in H1299 cells is dependent on activated p53, we have examined the apoptotic rate by Hoechst staining after p53 reconstitution. Figure 2c shows that p53 reconstitution significantly enhanced CDDP-induced apoptosis in the chemoresistant NSCLC cells H1299.

To further examine the role of p53 in EGFR suppression and in mediating CDDP-induced apoptosis in NSCLC, we knocked down p53 in the chemosensitive NSCLC cells, A549. As shown in Fig. 2d–f, p53 knockdown in A549 significantly decreased the apoptotic rate in response to CDDP. Also, it attenuated the activation of P-p53 and the suppression of P-EGFR induced by CDDP, suggesting that p53 is a key determinant in CDDP-induced apoptosis in NSCLC and that its action is in part a consequence of its modulation of EGFR activation.

### p53 is required for CDDP-inactivation of EGFR and induction of apoptosis in chemoresistant NSCLC cells following Akt inhibition

It is well established that Akt is a survival factor which mediates the signaling cascade of activated EGFR. As an oncoprotein, activated Akt is involved in diverse cell signaling and metabolic regulation. Although an elevated level of p53 has been reported to attenuate Akt pathway and to contribute significantly to enhanced cell invasion and higher metastatic potential [37], whether and how Akt regulates EGFR in NSCLC cells are still elusive.

**Fig. 3:**
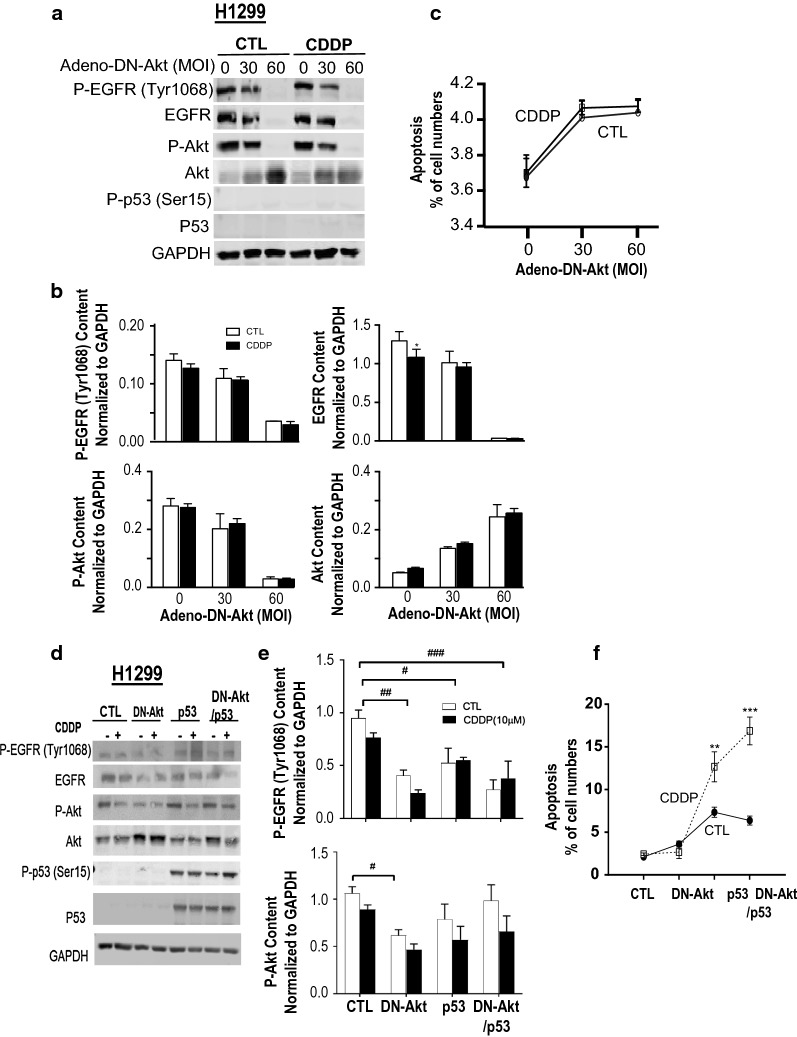
DN-Akt decreases p-/total EGFR expression levels and promotes ROS generation in chemoresistant NSCLC cells in response to CDDP. Chemoresistant H1299 cells were infected with adeno-DN-Akt or adeno-GFP (control; MOI = 0, 30 and 60; 5 h) and treated with CDDP (10 μM, 24 h). **a** Protein contents of [P-EGFR (Try 1068), EGFR, P-Akt, Akt, p-p53 (Ser 15), p53 and GAPDH (as loading control)] were detected by Western blot. **b** Statistical analysis and the quantification of indicated proteins were conducted. **c** Apoptosis was assessed by Hoechst staining. **d** H1299 Chemoresistant NSCLC cells were infected with adeno-GFP (control; MOI = 60; 5 h), adeno-DN-Akt (MOI = 60; 5 h), adeno-p53 (MOI = 0.25) alone, or together as indicated. **e** Statistical analysis and the quantification of indicated proteins were conducted [P-EGFR (Try 1068) and P-Akt]. **f** Apoptosis was assessed by Hoechst staining. Data were analyzed by two-way ANOVA followed by Bonferroni post hoc test (*P < 0.05, ***P < 0.001; CTL vs. CDDP; ^#^*P* < 0.05, ^##^*P* < 0.01, ^####^*P* < 0.0001; CTL vs. Adv-DN-AKT or Adv-p53)

To assess if Akt regulates EGFR in a positive feedback manner, chemoresistant H1299 cells were infected with the Adv-dominant negative Akt (Adv-DN-Akt) or Adv-GFP (as control; MOI = 0, 30, 60) and followed by treatment with CDDP (10 μM, 24 h). Successful Adv-DN-Akt expression was evident by increased total Akt content (WB). Adv-DN-Akt infection resulted in decreased P-Akt, total Akt and P-EGFR contents irrespective of the presence of CDDP, suggesting that Akt is also an upstream of EGFR and is associated with its activation (Fig. 3a, b). As expected, p53 expression was not evident in the p53 null chemoresistant H1299 cells (Fig. 3a). As shown in Fig. 3c, Adv-DN-Akt had no effect on CDDP-induced apoptosis in these chemoresistant H1299 cells. To assess if p53 is required for the induction of apoptosis and suppression of EGFR activation and Akt, H1299 cells were infected with Adv-DN-Akt (MOI = 60) and Adv-p53 (MOI = 0.25) alone or together followed by treatment with CDDP (10 μM, 24 h). Successful infection of Adv-DN-Akt and Adv-p53 was confirmed by increased content of Akt and p53 (WB), respectively (Fig. 3d). Adv-DN-Akt and Adv-p53 re-constitution together decreased the protein content of P-EGFR (Fig. 3d, e) and synergistically increased CDDP-induced apoptosis (Fig. 3f).

### Activation of Akt reduces ROS production and attenuates CDDP sensitivity by p53 suppression

**Fig. 4:**
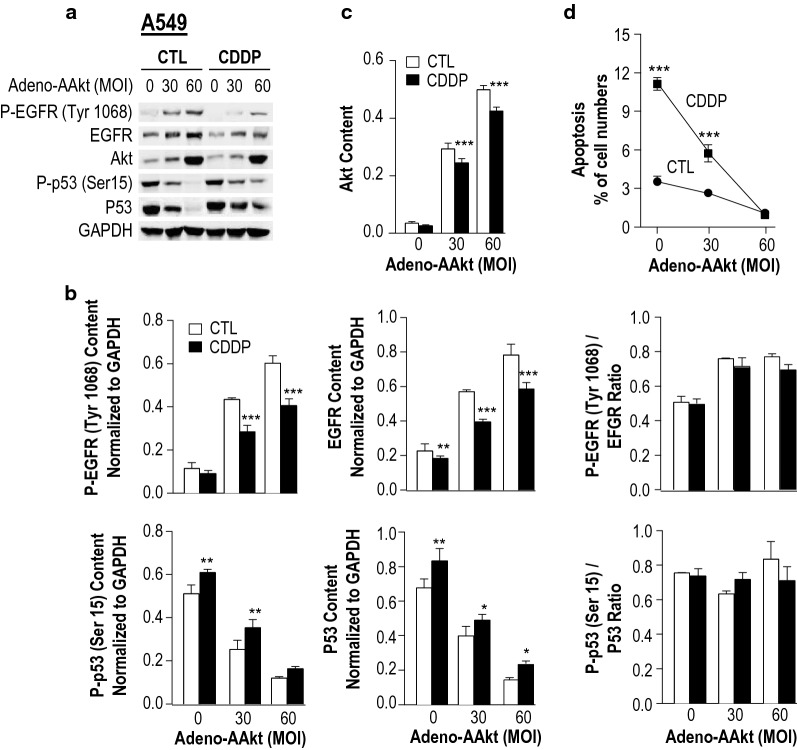
Activation of Akt down-regulates P-p53 and total p53 contents, up-regulates P-EGFR and EGFR contents and suppresses apoptosis in chemosensitive NSCLC cells. Chemosensitive A549 cells were infected with adeno-AAkt or control adeno-GFP (MOI = 0, 30, 60; 5 h) and cultured with CDDP (10 μM, 24 h). **a** P-EGFR (Try 1068), EGFR, Akt, P-p53 (Ser 15) and p53, and GAPDH (loading control) protein contents were examined by Western blot. **b** Quantitation and statistical analysis of protein contents [P-EGFR (Try 1068), EGFR, Akt, P-p53 (Ser 15), p53, and GAPDH] were conducted. **c** After AAkt overexpression and CDDP treatment, change in Akt content in A549 were determined. **d** Apoptosis was assessed by Hoechst staining. All data are expressed as mean ± SEM (n = 3); Data were analyzed by 2-way ANOVA and Bonferroni post hoc test (*P < 0.05, **P < 0.01, ***P < 0.001; CTL vs. CDDP)

To further demonstrate that Akt regulates EGFR content and participates in a positive feedback loop on EGFR signaling, chemosensitive A549 NSCLC cells were infected with a constitutively activated Akt (Adv-AAkt) or a control (Adv-GFP) and then treated with CDDP (10 μM, 24 h). Infection of A549 cells with Adv-AAkt led to a significant increase in total Akt content and attenuated the reduction in total and P-EGFR induced by CDDP (Fig. 4a, b), indicating that AAkt stabilizes total/P-EGFR contents in NSCLC cells. As shown in Fig. 4c, forced expression of AAkt attenuated the Akt activation induced by CDDP. Based on the notion that p53 mediates CDDP-induced apoptosis, we then determined whether the activation of Akt regulates p53 content and in turn CDDP-induced apoptosis. As expected, activated Akt decreased both total and P-p53 (Ser15) protein content and significantly protected chemosensitive A549 cells from CDDP-induced apoptosis (Fig. 4d), indicating that Akt is a survival factor in CDDP-induced apoptosis and its action is in part via modulation of p53 activation.

### Activation of P53 and Akt suppression are required for CDDP-induced elevation of ROS in NSCLC

**Fig. 5:**
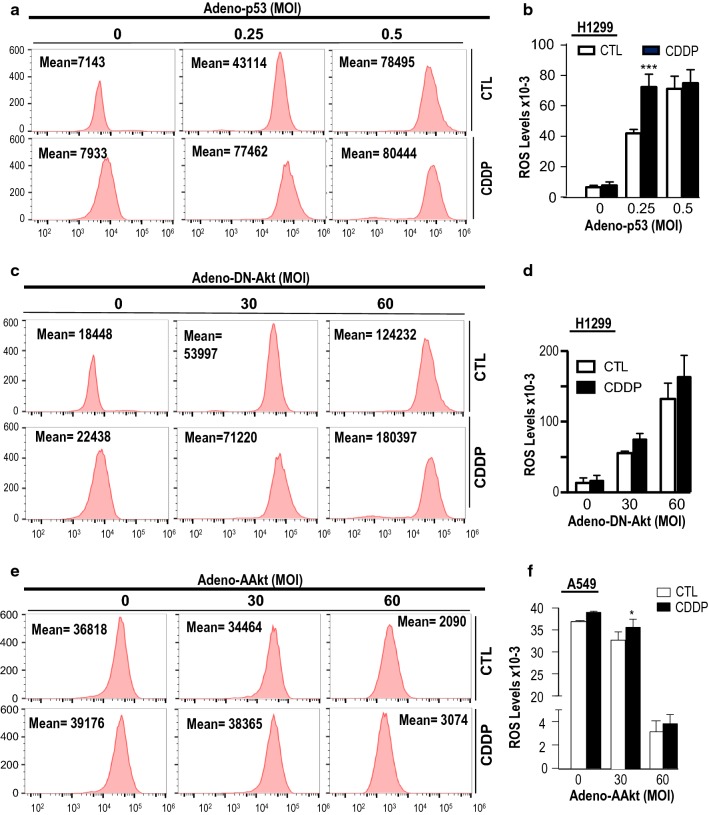
Effects of CDDP-induced ROS levels after p53 overexpression, dominant negative Akt overexpression and AAkt overexpression in chemoresistant (H1299) and chemosensitive (A549) NSCLC cells. **a** H1299 cells were infected with p53 adenovirus or adeno-GFP as control (MOI = 0, 0.25, 0.5 and 1; 5 h) and treated with CDDP (10 μM, 24 h). H1299 cells were stained with dichlorofluorescin diacetate (DCFDA) and ROS level was measured by flow cytometry. X axis represents DCFDA intensity and Y axis denotes cell count. Mean FL1 is equivalent of the area under the peak, representing fluorescence intensity. **c** H1299 cells were infected with DN-Akt adenovirus or adeno-GFP as control (MOI = 0, 30 and 60; 5 h) and treated with CDDP (10 μM, 24 h) and ROS was measured by flow cytometer. **e** A549 cells were infected with AAkt adenovirus or adeno-GFP as control (MOI = 0, 30 and 60; 5 h) and treated with CDDP (10 μM, 24 h). ROS was measured by flow cytometer. **b**, **d**, **f** Quantification and Statistical analysis of ROS production were conducted. Data, presented as mean ± SEM (n = 3), were analyzed by two-way ANOVA and Bonferroni post hoc test (*P < 0.05, **P < 0.01, ***P < 0.001; CTL vs. CDDP)

To investigate whether p53 regulates ROS production in NSCLC cells, we performed flow cytometric analysis of ROS accumulation. As shown in Fig. 5a and b, forced expression of Adv-p53 markedly enhanced ROS production in chemoresistant H1299 cells regardless of the presence of CDDP, suggesting increased p53 content alone or in combination with CDDP could stimulate a burst of cellular ROS. Interestingly, when Adv-p53 expression reached at maximal level (MOI = 0.5), no further increase in ROS was observed in the presence of CDDP. These results suggest that CDDP-induced ROS production is dependent on the activation of p53 and p53 is required for CDDP-induced apoptosis via suppression of EGFR and accumulation of ROS.

Previous studies have shown that elevated ROS levels suppress the EGFR/PI3K/Akt signaling pathway [38]. We further examined whether Akt can functionally regulate ROS production in chemoresistant H1299 cells. As shown in Fig. 5c and d, a loss of Akt significantly elevated ROS production by threefold when compared with the control. In addition, activated Akt reconstitution significantly reduced the basal level as well as CDDP-induced ROS production in chemosensitive A549 cells (Fig. 5e, f), Taken together, these findings suggest that Akt is important in the regulation of ROS production in NSCLC.

## Discussion

Lung cancer is the leading cause of cancer death worldwide and the development of chemoresistance has become a major hurdle to long term therapeutic success. Despite extensive research, the molecular mechanism of chemoresistance in lung cancer remains poorly understood. In the present study, we investigated the cellular and molecular mechanisms involved in NSCLC fate regulation, particularly in (1) the role and regulation of EGFR; (2) how the crosstalk between Akt and p53 regulates EGFR and ROS production, and (3) whether ROS is associated with CDDP-induced apoptosis. We have demonstrated for the first time that (a) CDDP down-regulates EGFR, activates p53 and increased ROS production in chemosensitive but not in chemoresistant NSCLC; (b) Akt confers resistance in NSCLC in part by activating EGFR and down-regulating p53 and ROS production; (c) p53 activation inhibits EGFR signaling and increased ROS production. Taken together, these results demonstrate that the interaction of the p53 and Akt feedback loops determine the fate of NSCLC cells and their CDDP sensitivity.

The mechanism of chemoresistance is multi-factorial and includes dysregulation of cell death and activated cell survival signaling pathways. The tumor suppressor p53 plays a pivotal role in the regulation of cancer cell fate in response to CDDP [39]. At least half of human cancers are associated with p53 mutations, resulting in the dysregulation of multiple signaling cascades including apoptotic pathways [40]. In contrast, Akt is a cell survival factor which inhibits apoptosis directly through the suppression of pro-apoptotic proteins and indirectly by inducing growth factor- and cytokine-mediated expression of anti-apoptotic protein [41]. Thus, p53 and Akt as well as their crosstalk are intimately involved in the regulation of apoptosis.

To our knowledge, this is the first report showing Akt activates EGFR. Substantial evidence reported that activation of EGFR results in the activation of the PI3K-Akt pathway and confers resistance to chemotherapy [42]. Although EGFR is frequently mutated in NSCLC, whether and how it affects chemosensitivity has not been fully elucidated. In the present studies, we have demonstrated that forced expression of an activated Akt in chemosensitive NSCLC cells resulted in increased both EGFR and p-EGFR protein contents in the absence of CDDP and suppressed CDDP-induced apoptosis. Moreover, expression of a dominant negative of Akt in chemoresistant NSCLC cells markedly down-regulated EGFR and p-EGFR and induced apoptosis. Taken together, our studies suggest that EGFR not only promotes Akt activation, but Akt in turn promotes EGFR signaling, forming a positive feedback circle within the EGFR-Akt axis. This is the first evidence showing this interplay between EGFR-Akt signal but precisely how Akt regulates EGFR requires further investigation.

Our findings demonstrate that Akt is involved in the regulation of p53 activation in NSCLC. We previously demonstrated that CDDP induced p53 phosphorylation at multiple serine sites in chemosensitive ovarian cancer cells although phosphorylation of p53 at the ser 15 and ser 20 was deemed an important determinant of chemosensitivity and that Akt suppressed these p53 activation processes [31, 39]. In NSCLC cells, the role of phosphorylated Ser15 site of p53 is critical in facilitating the transcriptional function of p53 and apoptosis [43]. It is also required in Chk2 activation in response to DNA damage [44].

In the present studies, we have shown that forced expression of an activated Akt in CDDP sensitive NSCLC resulted in a marked decrease in both p53 and P-p53 contents regardless of the presence of CDDP (Fig. 4). In addition, activation of Akt suppressed CDDP-induced apoptosis in a p53-dependent-manner. Taken together, these findings support the notion that Akt is involved in the regulation of p53 contents and its mediated apoptosis in NSCLC.

Although *EGFR* and *TP53* genes are frequently mutated in NSCLC, their role and interaction in regulating chemosensitivity have not been fully elucidated. Moreover, whether p53 is involved in the regulation of EGFR in NSCLC is unclear. Although p53 has been shown to negatively regulate PI3K gene transcription [45], whether p53 controls EGFR activation and chemosensitivity in NSCLC, is not known. In the present study, reconstitution of p53 null cells with wild type p53 down-regulated EGFR and p-EGFR content, and increased the P-EGFR/EGFR ratio in the presence of CDDP, suggesting that p53 is required for EGFR down-regulation and CDDP-induced apoptosis (Fig. 2).

Elevated production of ROS function is an effector of apoptosis in cancer cells [46] and, depending on its intracellular level, ROS could play a dual role. At low intracellular level, ROS activation is required for stimulation of various growth factors and cytokines, and it regulates downstream signaling pathways, leading to specific cellular functions [47]. When the cells are stressed by external stimuli like CDDP, ROS concentration is markedly increased, inhibiting cell cycle progression and inducing apoptosis [48]. It has been reported that CDDP accumulates in mitochondria and forms adducts with mitochondrial DNA, leading to the impaired synthesis of proteins involved in electron transport chain and increased intracellular ROS level [49, 50]. However, the mechanism of CDDP-induced generation of ROS and their contribution to cisplatin cytotoxicity in cancer cells is poorly understood.

We and others have demonstrated that CDDP induces mitochondrial mediated-apoptosis that is p53-dependent [7, 51]. However, the role of mitochondrial ROS production in chemoresistant NSCLC is not known. In the present studies, CDDP down-regulated EGFR and increased ROS production and CDDP-induced ROS generation appeared to be p53-mediated (Fig. 5). Moreover, Akt activation in chemosensitive NSCLC cells (A549) significantly reduced ROS production and conferred CDDP resistance. Taken together, these findings suggest that CDDP induces apoptosis in chemosensitive NSCLC through p53-mediated ROS production, a process also involving EGFR down-regulation. In addition, Akt confers CDDP resistance through EGFR activation and ROS down-regulation. Although this study only investigates intracellular ROS level, we need to further investigate the role of mitochondrial ROS in chemoresistance.

In conclusion, by in vitro pharmacological approach and mechanistic studies on the relationship between EGFR, Akt, p53 and ROS in sensitive and resistant NSCLC cells, we have demonstrated that p53 sensitizes chemoresistant cells to CDDP by suppressing EGFR signaling and promoting ROS generation. We also observed that Akt confers resistance through a yet undetermined positive feedback mechanism on EGFR signaling and down-regulating ROS production, thus suppressing apoptosis in chemoresistant NSCLC. Previous studies have demonstrated that intracellular ROS production is higher in cancer cells than normal cells and the presence of ROS can promote cell survival. However, our studies found that chemosensitive A549 cells showed higher level of ROS production compared with chemoresistant 1299 cells and CDDP causes ROS production in cancer cells leading to apoptosis, but this response is attenuated in chemoresistant cells. Therefore, elevating ROS in cancer cells can be considered as potential therapeutic strategy for chemoresistant cancer.

**Fig. 6:**
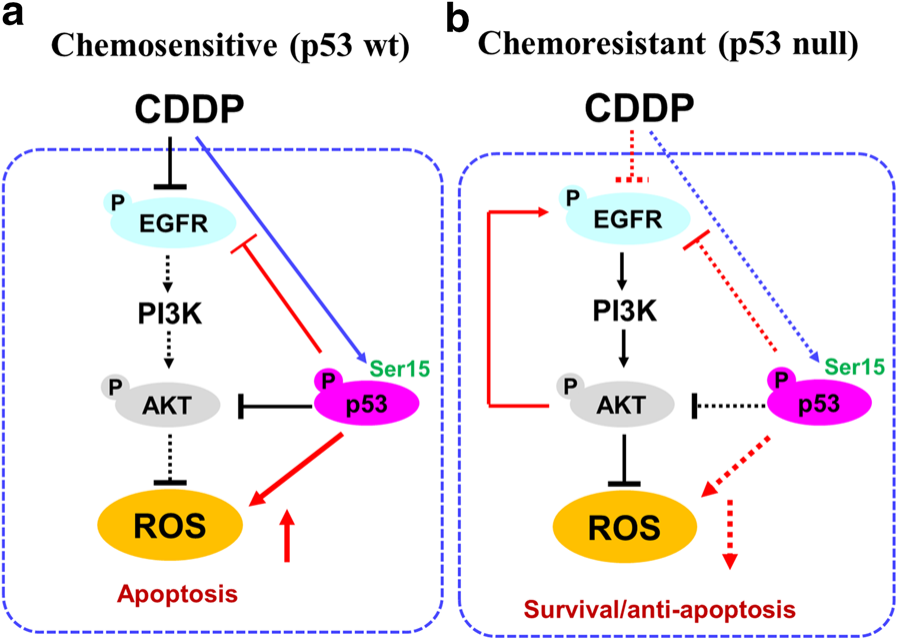
Hypothetical model illustrating the role and regulation of EGFR and ROS production in NSCLC cells. **a** In chemosensitive NSCLC cells: (1) CDDP treatment leads activation p53 at the site of Ser15; (2) this impairs the activation of EGFR and its downstream signaling cascades, PI3K and Akt; (3) Decreased Akt and activated p53 contribute together increase in CDDP-induced ROS production. **b** In chemoresistant NSCLC cells, (1) CDDP failed to activate p53; (2) EGFR can be activated and promotes its downstream PI3K-Akt signaling cascade; (3) Due to the absence of activated p53 and hyperactivated Akt, CDDP-induced ROS is decreased and in turn apoptosis is impaired. Solid line indicates activated molecular action whereas dashed line indicates suppressed signaling. Arrows indicates direct action whereas blocked line indicates inhibition

To facilitate future investigation on the molecular basis of chemoresistance in NSCLC, we propose the following hypothetical model (Fig. 6). In chemosensitive cells, p53 suppresses EGFR signaling and induces ROS production in response to CDDP. In chemoresistant cells, Akt suppresses CDDP-induced apoptosis by decreasing p53 and ROS levels as well as promoting EGFR signaling in NSCLC. While the present studies significantly advance the current understanding of the molecular basis of chemoresistance in NSCLC, whether these in vitro findings are applicable in vivo remains to be tested. It will be of particular interest to examine if the EGFR inhibitor and exogenous p53 re-constitution could sensitize chemoresistant NSCLC cells to CDDP in a mouse xenograft model and if this phenomenon is associated with down-regulation EGFR signaling and increased ROS production.

## Conclusion

In this study, we unraveled a novel mechanism how p53 suppresses PI3K/EGFR/Akt by modulating reactive oxygen species (ROS) in NSCLC. In chemosensitive NSCLC, p53 plays a pivotal role in sensitizing cells to CDDP by elevating intracellular ROS level and suppressing EGFR/PI3K/AKT signaling. Conversely, in chemoresistant NSCLC, we identified the novel mechanism that Akt in turn promotes the function of EGFR via a positive feedback loop, leading to the suppression of ROS production and chemoresistance. With mechanistic and pharmacological approaches, this study advances the current understanding of chemoresistance in NSCLC.

## Additional file

**Additional file 1.** Additional tables and figure.

## Data Availability

Not applicable.

## Abbreviations

CDDP: Cis-diaminedichloroplatinum (Cisplatin)
EGFR: epidermal growth factor
NSCLC: non-small cell lung cancer
PI3K: phosphatidylinositol 3-kinase
ROS: reactive oxygen species
TKI: tyrosine kinase inhibitor
