# Supplementary material for: p53 sensitizes chemoresistant non-small cell lung cancer via elevation of reactive oxygen species and suppression of EGFR/PI3K/AKT signaling
Source: Cancer Cell Int. 2019 Jul 19;19:188. doi: 10.1186/s12935-019-0910-2 (PMC6642601; doi:10.1186/s12935-019-0910-2)
Supplement: Supplementary file 1 — Additional file 1. Additional tables and figure. [file 12935_2019_910_MOESM1_ESM.pptx]

## Slide 1
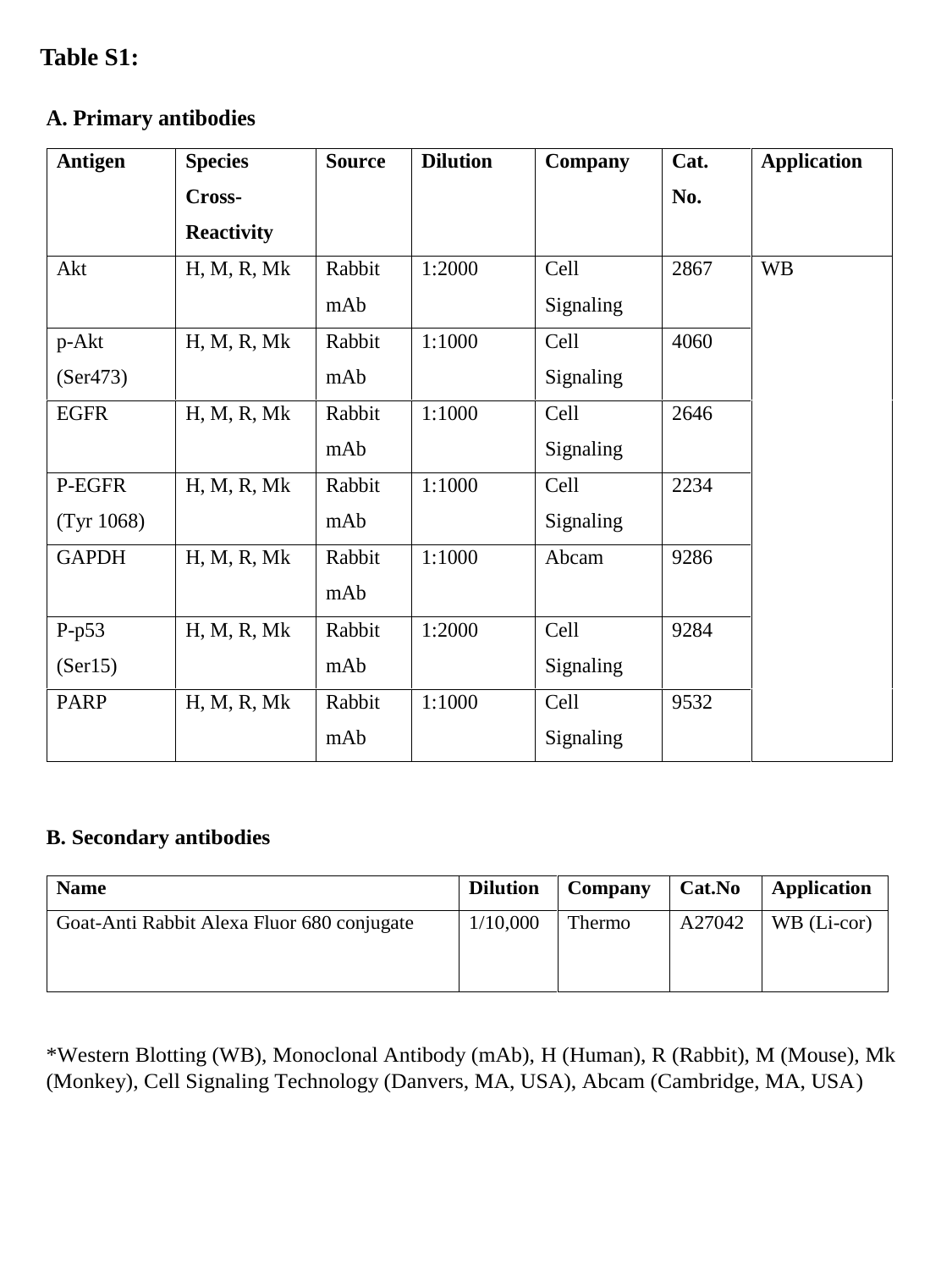

Table S1:

## Slide 2
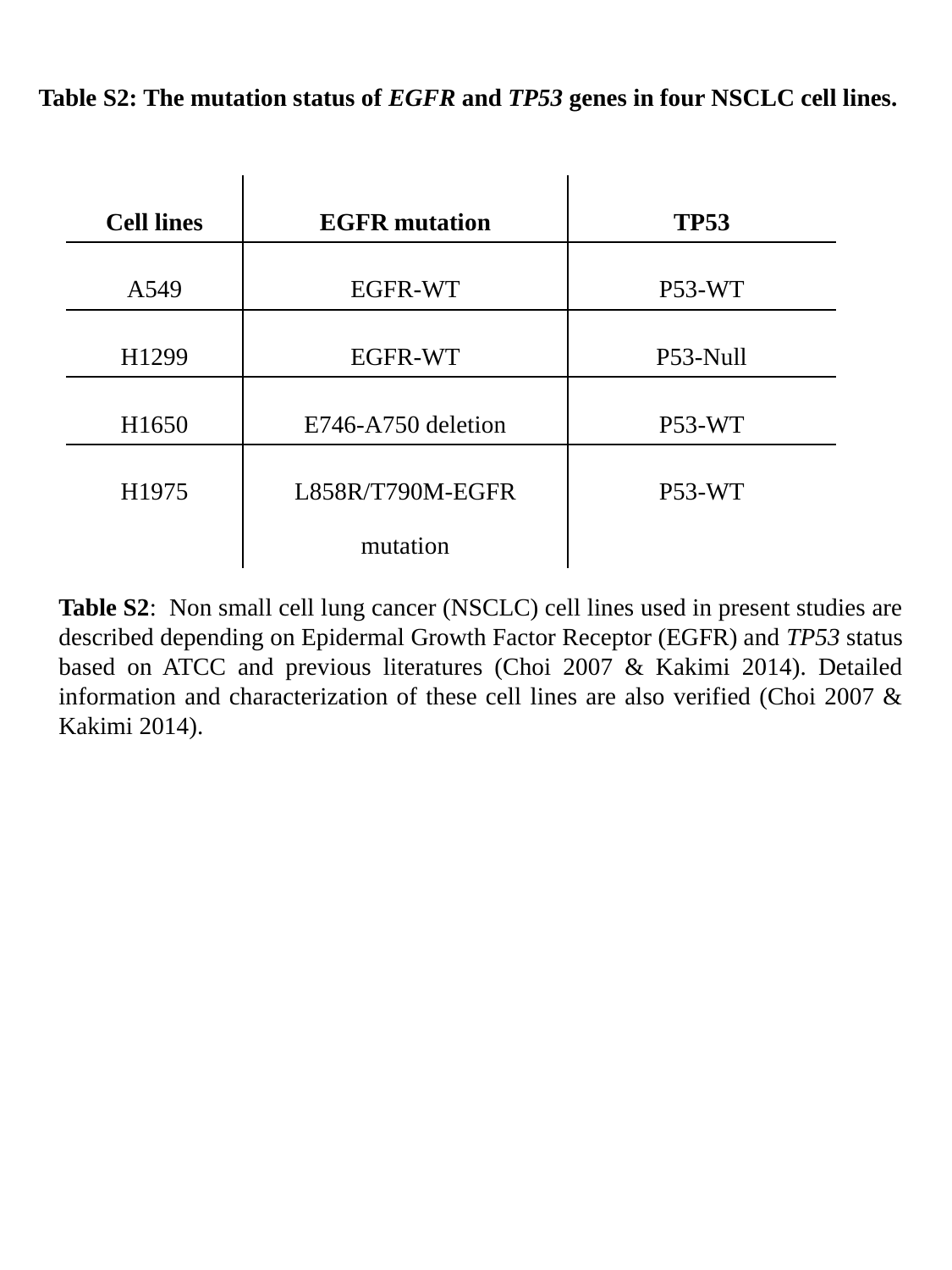

Table S2: The mutation status of EGFR and TP53 genes in four NSCLC cell lines.
| Cell lines | EGFR mutation | TP53 |
| --- | --- | --- |
| A549 | EGFR-WT | P53-WT |
| H1299 | EGFR-WT | P53-Null |
| H1650 | E746-A750 deletion | P53-WT |
| H1975 | L858R/T790M-EGFR mutation | P53-WT |
Table S2: Non small cell lung cancer (NSCLC) cell lines used in present studies are described depending on Epidermal Growth Factor Receptor (EGFR) and TP53 status based on ATCC and previous literatures (Choi 2007 & Kakimi 2014). Detailed information and characterization of these cell lines are also verified (Choi 2007 & Kakimi 2014).

## Slide 3
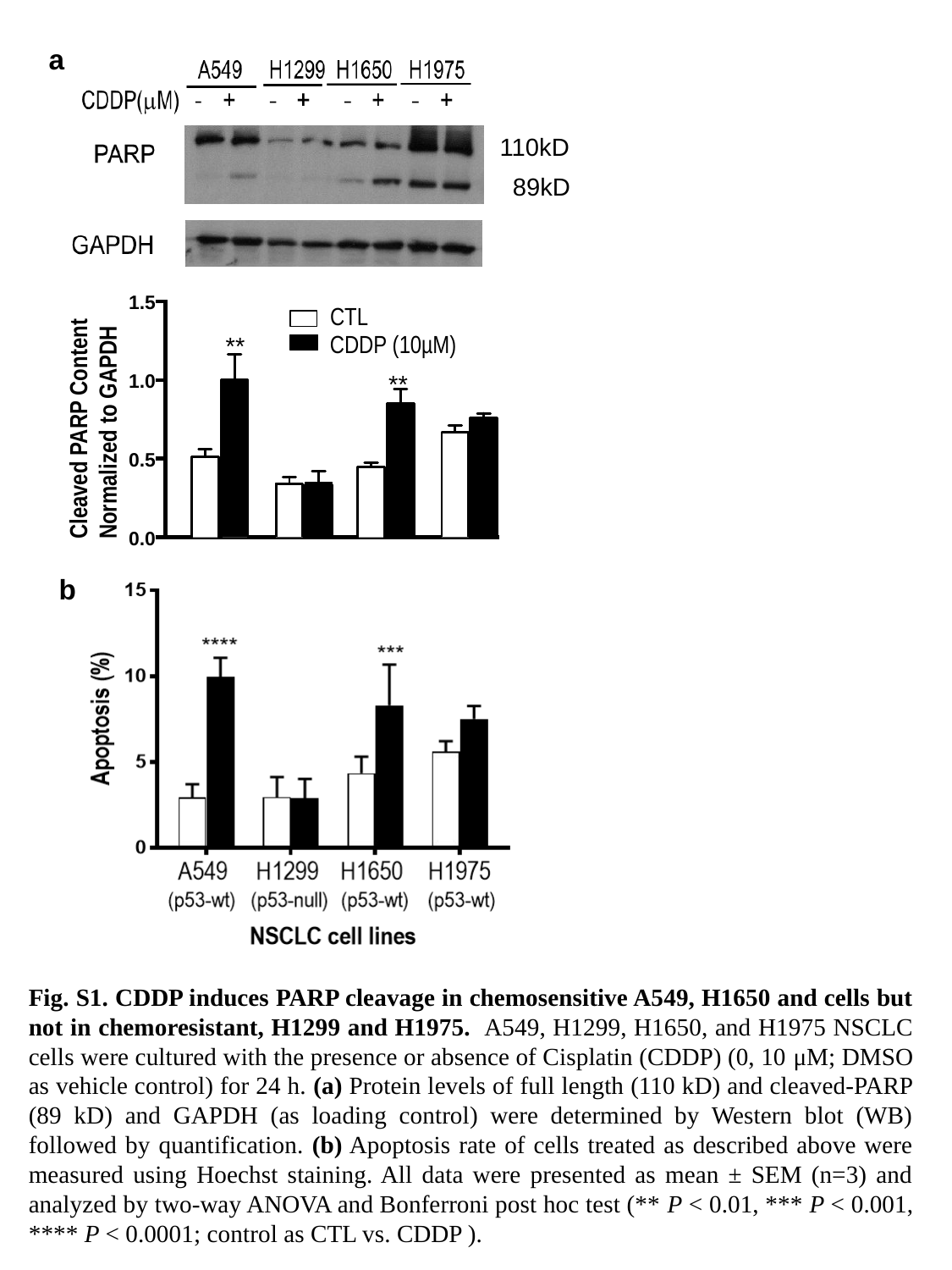

a
110kD
 89kD
1.5
CTL
CDDP (10µM)
**
1.0
**
Cleaved PARP Content
Normalized to GAPDH
0.5
0.0
b
Fig. S1. CDDP induces PARP cleavage in chemosensitive A549, H1650 and cells but not in chemoresistant, H1299 and H1975. A549, H1299, H1650, and H1975 NSCLC cells were cultured with the presence or absence of Cisplatin (CDDP) (0, 10 μM; DMSO as vehicle control) for 24 h. (a) Protein levels of full length (110 kD) and cleaved-PARP (89 kD) and GAPDH (as loading control) were determined by Western blot (WB) followed by quantification. (b) Apoptosis rate of cells treated as described above were measured using Hoechst staining. All data were presented as mean ± SEM (n=3) and analyzed by two-way ANOVA and Bonferroni post hoc test (** P < 0.01, *** P < 0.001, **** P < 0.0001; control as CTL vs. CDDP ).
